# Evaluation of Five Evapotranspiration Products and Driving Factors over the Plateau and Plains of China

**DOI:** 10.3390/s26144572

**Published:** 2026-07-19

**Authors:** Wei Fu, Bo-Hui Tang, Changjun Deng, Xinming Zhu, Zhongxi Ge, Junyi Chen

**Affiliations:** 1 Yunnan Key Laboratory of Quantitative Remote Sensing/Yunnan International Joint Laboratory for Integrated Sky-Ground Intelligent Monitoring of Mountain Hazards, Faculty of Land Resources Engineering, Kunming University of Science and Technology, Kunming 650093, China; fuwei@stu.kust.edu.cn (W.F.); dengchangjun@stu.kust.edu.cn (C.D.); zhuxinming0330@163.com (X.Z.); zhongxige@kust.edu.cn (Z.G.); junyichen@kust.edu.cn (J.C.); 2 Southwest United Graduate School, Kunming 650092, China; 3 State Key Lab of Resources and Environmental Information System, Institute of Geographic Sciences and Natural Resources Research, Chinese Academy of Sciences, Beijing 100101, China

**Keywords:** evapotranspiration, remote sensing, plateau, plain, driving factors

## Abstract

**Highlights:**

**What are the main findings?**

- PML_V2 and ETMonitor outperform other products in accuracy and sensitivity over the Tibetan Plateau, while GLASS exhibits superior spatial continuity across the Yunnan–Guizhou Plateau and plains.
- Ecosystem-specific controls of ET are identified: thermal and vegetation factors drive the TP, contrasting with thermal-moisture interactions on the YGP and synergistic controls in the plains.

**What are the implications of the main findings?**

- The distinct region-specific mechanisms necessitate the development and application of tailored hydrological models that incorporate local thermal, moisture, and vegetation regimes.
- Quantifying ET discrepancies between plateaus and plains provides a critical scientific basis for optimizing remote sensing algorithms and enhancing regional water resource management.

**Abstract:**

Evapotranspiration (ET) plays a pivotal role in the water and energy cycles, yet the accuracy of ET products and their driving mechanisms varies significantly across complex topographies. This study systematically evaluates five ET products and investigates their driving mechanisms across the Tibetan Plateau (TP), the Yunnan–Guizhou Plateau (YGP), and the lowlands of China. The results show that, while ET products generally achieve higher accuracy in lowlands, the PML_V2 and ETMonitor demonstrate superior performance and sensitivity in the challenging plateau environments. Distinct regional heterogeneity was observed: the TP is dominated by thermal radiation factors, the YGP is constrained by both thermal and moisture conditions, and the lowlands are driven by the synergistic interaction of thermal, moisture, and vegetation factors. Unlike previous studies, which often treat these regions homogeneously, this work highlights the critical necessity of region-specific model selection to mitigate uncertainties arising from terrain changes. These findings advance understanding of ET discrepancies, facilitate the selection of appropriate ET products, promote model optimization in ET retrieval research, and enhance water management and hydrological modeling under varying topographical conditions.

## 1. Introduction

In the global water cycle, approximately 60% of annual precipitation (Prec) returns to the atmosphere through evapotranspiration (ET); in arid regions, this proportion can reach 90% [1,2]. Accurately evaluating ET products with varying spatial–temporal resolutions is essential for selecting the ET product with the highest accuracy for a specific region, which is vital for regional hydrological research [3,4,5].

The methods of actual ET observation and estimation generally fall into two categories: direct measurement and indirect retrieval [6,7,8]. In situ ET measurement techniques represent the most direct and accurate approach for obtaining ET observations [9], including lysimeters [10], eddy covariance (EC) systems [11,12,13], large-aperture scintillometers (LASs) [14], and the Bowen ratio method (BR) [15]. However, these in situ measurement approaches are limited by several key constraints, including high upfront equipment expenses, sparse spatial coverage, and a restricted observational footprint. Consequently, these approaches cannot support the requirements of spatially continuous ET estimation at regional or global scales [16,17]. It is encouraging that numerous ET products have been developed in recent years using indirect methods, such as remote sensing (RS)-based retrieval and reanalysis products, which have helped address the limitations of in situ monitoring and established a robust data foundation for regional and global hydrological analysis. These existing ET products are typically classified into four categories: (1) RS-based products, such as the MODIS Global ET Project (MOD16A2GF Version 6.1, denoted by MOD16) [18,19], and the Simplified Surface Energy Balance Operational ET (SSEBop) [20]; (2) reanalysis-based products, such as the fifth-generation ECMWF atmospheric reanalysis (ERA5) [21]; (3) data-driven model products, like the Global Land Surface Satellite (GLASS) ET product [22]; and (4) water balance-based products [6,23]. RS-based ET products are well suited to regions with sparse meteorological data and records and generally offer higher spatial and temporal resolutions. However, their accuracy is often significantly compromised by cloud cover and gap-filling [24,25,26,27,28]. For instance, the MOD16 product excludes water-body evaporation and nighttime ET, yet exhibits substantial data gaps in mountainous areas. Reanalysis-based ET products are characterized by relatively coarse spatial resolutions but high temporal resolutions and extended temporal coverage. Nevertheless, limitations in their model structures often hinder the accurate representation of critical physical processes [21,29]. Data-driven ET products synthesize multiple ET estimates to reduce the uncertainty associated with any single product. However, they are encumbered by complex structures, high data and computational demands, a lack of standardized protocols for algorithm fusion, and diminished physical interpretability [22,30,31]. Water balance-based products are derived using surface water budget and atmospheric water balance methods. However, they primarily capture domain-averaged ET over monthly or annual timescales, often failing to capture the pronounced spatiotemporal heterogeneity of ET [32,33,34]. Collectively, these existing products do not adequately account for topographic impacts on ET in mountainous regions; therefore, it is necessary to develop ET products that explicitly incorporate topographic effects [35,36].

However, uncertainties in ET products persist across regions, particularly within diverse climatic and topographically complex zones [37,38,39,40]. These uncertainties largely stem from substantial disparities in input data, model frameworks, and parameter settings employed across various ET estimation methodologies [41,42,43]. Consequently, it is essential to evaluate existing ET products prior to quantifying ET and exploring spatiotemporal variability patterns across different regions [44]. It is noteworthy that EC-based ground-level measurements of ET have served as a benchmark dataset for verifying the accuracy of ET products at the point scale [26,45,46]. Despite limitations such as energy imbalance and spatial mismatches in ground-based measurements, EC remains the most direct and effective means of validation. The water balance method constitutes another approach utilized for long-term ET evaluation at regional basin scales [23,47,48]. However, this method is constrained by the accuracy of water balance input parameters over short time scales, such as terrestrial water storage, precipitation, and runoff data within a watershed [38]. The triple collocation (TC), three-cornered hat (TCH) [16,49,50], and extended collocation method (ECM) [51,52] are also widely applied for the validation and evaluation of various ET products. While these methods are effective for estimating the relative uncertainty of ET products, they are limited in their ability to quantify the absolute discrepancy between estimated and actual ET. For example, Tang et al. [6] validated and analyzed 25 global ET datasets using EC and catchment water balance data, revealing the characteristics of global annual average ET and its spatiotemporal variations. However, they did not explore the differences between these products in mountainous and plain areas or investigate the key drivers of different products. Zuo et al. [48] validated six ET products for China using the three aforementioned methods, with results showing that GLEAM and Penman–Monteith–Leuning V2 (PML_V2) performed better at both point and catchment scales, whereas GLASS displayed lower uncertainty. The limited number of EC sites selected in this study to evaluate the performance of ET products is insufficient to elucidate how each product performs in different ecosystems.

Mountainous areas worldwide account for approximately 23% of the total land area, while mountainous areas in China account for approximately 67% of the country’s total land area [35]. Mountain regions play a crucial role in delivering essential ecosystem services, such as the water supply, the preservation of ecosystem biodiversity, and the regulation of regional and even global climate [53]. Studies by Liu et al. [23] have shown that different ET products exhibit significant discrepancies across altitude gradients. However, there has been little research into the performance and spatiotemporal variations of ET products in the plateau mountainous areas. Zheng et al. [47] evaluated 22 ET products across the Tibetan Plateau (TP) at both the in situ and basin scales. However, the study did not compare the accuracy of different ET products across different ecoregions, nor did it examine differences in the drivers of these ET products.

Accurate estimation of ET in mountainous areas remains a formidable challenge due to heterogeneous landscapes, complex terrain, and diverse climatic conditions characterized by vertical gradients and significant thermal fluctuations [36]. Most current assessments of ET product accuracy focus on homogeneous regions, often disregarding topographic differences or relying on sparse ground station data. Consequently, there is a paucity of research investigating the comparative performance of ET products and their underlying driving factors across distinct topographies, specifically between plateaus and plains. This highlights the need for more comprehensive studies that take into account the unique characteristics of different terrains and expand the scope of ground station data applications to provide more accurate assessments and application guidelines for ET products across various landforms.

To fill those gaps, the primary objectives of this paper are to (1) investigate the spatiotemporal variability of ET and assess the performance of five ET products using EC observations in plateau and lowland areas; (2) explore the spatiotemporal distribution differences of various ET products in plateau and plain regions; and (3) analyze the key driving factors for the five ET products across plateau and plain regions to diagnose the potential responses presented by different products. Section 2 describes the study area and datasets. Section 3 presents the evaluation methodology. Section 4 presents the results, specifically: the differences in ET among the TP, the YGP, and other regions of China based on EC data; and the performance disparities, spatiotemporal distributions, and driving factors over plateau and plain regions. Discussions and conclusions are provided in Section 5 and Section 6, respectively. The contribution of this study lies in elucidating the differences in ET between plateau and lowland regions and the mechanisms driving these differences, and in providing recommendations for selecting appropriate products for hydrological analysis across different topographic and ecological scales.

## 2. Study Area and Dataset

### 2.1. Study Area

**Figure 1:**
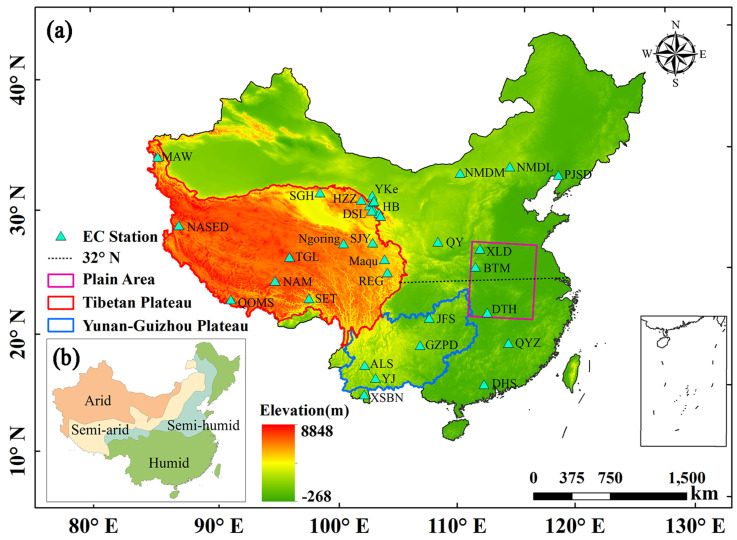
Overview of the study area: (**a**) the geographical distribution of the study area, including EC stations, sub-study areas, and elevation; (**b**) the four climate zones of China.

In this study, we selected China (3°30′00″~53°33′47″ N, 73°29′50″~135°2′30″ E) as the study area (Figure 1). China covers approximately 9.6 million km^2^ and features topographically complex landscapes, including basins, hills, plateaus, and plains [48]. Given its extensive land area, the country encompasses a variety of climate types, from arid and semi-arid regions to semi-humid and humid ones (Figure 1b). Specifically, we investigated the differences in ET between plateau and lowland areas using 34 EC data distributed across the TP (16 stations), the Yunnan–Guizhou Plateau (YGP; 5 stations), and other regions of China (13 stations); additionally, we evaluated the performance of five ET products across these regions. To compare the performance of the five ET products across the TP, YGP, and lowland regions, the EC stations located in other parts of China were stratified into two groups based on the 32° N latitude line. This parallel approximates the Qinling Mountains–Huai River line, a significant geographical boundary that separates northern and southern China and distinguishes between semi-humid and humid climates. These sub-regions were defined as Region I (north of 32° N, comprising 10 stations) and Region II (south of 32° N, comprising 3 stations). Subsequently, the TP, YGP, and a plain area—delineated as a rectangular zone covering the Huai River basin and parts of Yangtze River’s middle and lower reaches (111–118° E, 29–35° N, designated as the HYP)—were selected as specific sub-study areas to investigate the spatiotemporal variations and driving factors of the five ET products across plateau and plain terrains. The spatial distributions of the study areas and EC sites are illustrated in Figure 1a.

### 2.2. Datasets

#### 2.2.1. Eddy Covariance Data

The EC data for this research were sourced from the ChinaFLUX network (http://www.chinaflux.org, accessed on 30 April 2026), the Heihe Watershed Allied Telemetry Experimental Research (HiWATER) network [54], and the Institute of Tibetan Plateau Research, Chinese Academy of Sciences (ITPCAS) [55]. Essential information regarding the EC datasets is presented in Table 1. High-quality, daily or monthly gap-filled ET data were readily available, for instance, from ChinaFLUX and HiWATER [54], and were thus directly adopted. For EC supplying half-hourly or hourly data, specifically ITPCAS, these data were compiled into the monthly ET dataset [55].

#### 2.2.2. ET Products

In this study, five RS-based ET products (MOD16, GLASS, PML_V2, SSEBop, and ETMonitor) were selected, all with spatial resolutions coarser than 1000 m and derived from different algorithms. A summary of the basic information regarding these datasets is described in Table 2. Considering that the overlap period for the five ET products spans 2003–2020, we selected it as the study period (Table 2). MOD16 is a global land-surface ET dataset generated using the modified Penman–Monteith (PM) model, which combines Aqua and Terra satellite data with meteorological reanalysis data and MODIS data (https://www.ntsg.umt.edu, accessed on 30 April 2026). The product provides four components: ET, latent heat (LE), potential ET, and potential latent heat flux [18]. PML_V2 integrates a biophysical model with a canopy stomatal conductance model within the PM equation to calculate gross primary productivity (GPP) and ET [56]. The GLASS utilizes Bayesian model averaging (BMA) to synthesize five process-based ET models: MS-PT, RRS-PM, MODIS, UMD-SEMI, and PT-JPL, whose data sources are available at https://glass.hku.hk (accessed 30 April 2026) [22]. By applying the satellite psychrometry principle, the SSEBop directly computes ET without requiring the determination of other surface energy balance components [20,25]. SSEBop ET is provided by the US Geological Survey (USGS) Famine Early Warning System Network (FEWS NET) (https://earlywarning.usgs.gov, accessed on 30 April 2026). The ETMonitor model employs a multi-process parameterization scheme that accounts for land-surface energy, moisture, and physiological processes of vegetation. The ET calculated by ETMonitor for different land cover types (LCTs) encompasses individual assessments of soil evaporation, plant transpiration, and intercepted canopy evaporation for mixed land cover consisting of vegetation and soil; calculation of water surface evaporation for dynamic water bodies; and the calculation of snow and ice sublimation for snow and ice surfaces. The ET for a pixel is the sum of evaporation, transpiration, and sublimation [57].

Before assessment, the ET products, which vary in temporal and spatial resolution, were aggregated monthly from their original temporal scales to yield a cumulative ET. For daily-resolution ET datasets, e.g., PML_V2 and ETMonitor for 2020, monthly ET was obtained by simple summation. For datasets with an 8-day resolution, such as MOD16 and GLASS, the daily average ET is calculated from existing ET data for a given month, and this daily average is then multiplied by the total number of days in that month to estimate monthly ET. In addition, given inconsistencies in the spatial resolution of the five ET products, ArcGIS 10.7 was used to project, resample using the bilinear interpolation method, and clip all raster datasets to a 500 m × 500 m resolution.

#### 2.2.3. Auxiliary Dataset

The auxiliary datasets collected include the China Meteorological Forcing Dataset v2.0 (CMFD), precipitation data, MODIS, GLASS, Monthly mean land-surface temperature (Ts), and sunshine duration (SSD). These auxiliary datasets were used to investigate driving factors of the five ET products across the three sub-regions. The CMFD, a gridded dataset with high spatial resolution (0.1°×0.1°), integrates remotely sensed products, reanalysis datasets, and field data from 1951 to 2024 [58]. This dataset provides meteorological variables, including air temperature (Ta), atmospheric pressure (Pa), wind speed (WS), relative humidity (RH), downward shortwave radiation (SWD), and downward longwave radiation (DLR), available at 3-h, daily, monthly, and annual resolutions. The dataset is accessible at https://dx.doi.org/10.11888/Atmos.tpdc.302088 (accessed on 30 April 2026). Monthly precipitation data with a spatial resolution of 1 km were sourced from the Qinghai–Tibet Plateau Research Institute under the Chinese Academy of Sciences (https://data.tpdc.ac.cn/, accessed on 30 April 2026) [59].

The MODIS primarily comprises MCD43C3 for deriving monthly average albedo (1-day, 500 m) and MOD13A2 for deriving monthly average normalized Difference Vegetation Index (NDVI) (16-day, 1 km); these data are accessible via Google Earth Engine (GEE). In this study, the 8-day leaf area index (LAI) (500 m) and Rn (1-day, 0.05°) products from the GLASS dataset were used to investigate the driving factors of five ET products [60,61]. Access to the GLASS dataset is available through the provided link (https://glass.hku.hk/, accessed on 30 April 2026). A global dataset of monthly mean LST (MMLST) at a 1 km × 1 km resolution was created for the period 2003–2020 [62]. The 1-day, 4 km resolution SSD dataset was from Zhang et al. [63]. Due to inconsistencies in the spatial resolution of the aforementioned data, ArcGIS 10.7 was used to project and resample all raster datasets to a 500 m × 500 m resolution.

## 3. Methodology

### 3.1. Conversion from Monthly LE Data to Equivalent ET

Given some EC observations and ET products that provide LE flux, expressed in units of (W/m^2^). In this research, we initially transformed the monthly LE data (W/m^2^) into equivalent ET (mm/month) [23].

(1) ET=LE/λ

where λ represents the latent heat of vaporization (2.45 MJ/kg).

### 3.2. Statistical Evaluation Metrics

This study selected the Pearson correlation coefficient (*R*), the coefficient of determination (*R*^2^), *RMSE*, and *bias* as statistical metrics to evaluate the *ET* products. The definitions of these evaluation metrics are as follows:

(2) $$R=\frac{\sum_{i=1}^{n}\left({ET}_{p,i}-\bar{{ET}_{p}}\right)\left({ET}_{o,i}-\bar{{ET}_{o}}\right)}{\sqrt{\sum_{i=1}^{n}{\left({ET}_{p,i}-\bar{{ET}_{p}}\right)}^{2}}\sqrt{\sum_{i=1}^{N}{\left({ET}_{o,i}-\bar{{ET}_{o}}\right)}^{2}}}$$

(3) $$R^{2}=1-{\sum_{i=1}^{n}\left[{ET}_{p,i}-{ET}_{o,i}\right]}^{2}/\sum_{i=1}^{n}{\left({ET}_{o,i}-\bar{{ET}_{o}}\right)}^{2}$$

(4) $$RMSE=\sqrt{\frac{1}{n}\sum_{i=1}^{n}{\left({ET}_{p,i}-{ET}_{o,i}\right)}^{2}}$$

(5) $$Bias=\frac{1}{n}\sum_{i=1}^{n}\left({ET}_{p,i}-{ET}_{o,i}\right)$$

where the *ET_p_* is the ET from various products (mm/month); *ET_o_* is the ET derived from EC; the subscripts *i* and *n* are the index of the ith sample and the total number, respectively; $\bar{{ET}_{p}}$ and $\bar{{ET}_{o}}$ is the average of the ET products and observed ET, respectively.

### 3.3. Geographical Detector Model

Geographical detectors are grounded in the measurement of factor force indicators and function as statistical tools for evaluating spatially stratified heterogeneity and its influencing factors [64,65,66]. The calculation of the q-value is as follows:

(6) $$q=1-\frac{\sum_{i=1}^{L}N_{i}\sigma_{i}^{2}}{N\sigma^{2}}$$

where *i* is the stratification of the response variable *Y* or driving factor *X*; *N_i_* and *N* are the number of units in the *i-th* stratum and the total sample size, respectively; $\sigma_{i}^{2}$ and $\sigma^{2}$ are the variances of *Y* in stratum *i* and the entire study area, respectively.

In this study, the five ET products examined can be categorized into three groups: the PM-based approach (MOD16, PML_V2, and ETMonitor), the data-driven method (GLASS), and the surface energy balance approach (SSEBop). Based on the key input parameters of the models mentioned above, this study selected the following variables for analysis: (1) radiation-related variables (Rn, DLR, and SWD); (2) land-surface characteristics and vegetation variables (albedo, LAI, and NDVI); and (3) meteorological variables (Ts, Ta, Pa, RH, WS, and vapor pressure deficit, VPD). Given that the ET process is significantly influenced by precipitation, precipitation was also included as a driving factor. Additionally, since SSD significantly impacts total solar radiation and vegetation growth, it was also selected as an explanatory variable in this study. The VPD is determined by the difference between the saturated vapor pressure and the actual vapor pressure at a Ta, as detailed in [67].

## 4. Results

### 4.1. ET Differences Between the TP, YG, and Other Regions of China Based on EC Data

**Figure 2:**
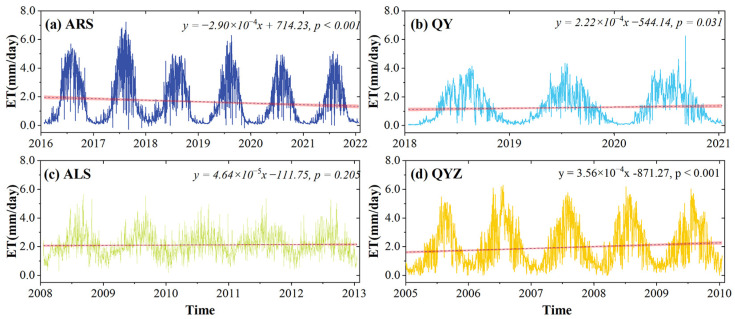
Differences in the temporal variation of daily ET between plateau and plain regions: (**a**) ARS at TP; (**b**) QY at Region I; (**c**) ALS at YGP; (**d**) QYZ at Region II. The dashed lines represent the fitted trends, and the shaded areas denote the 95% confidence interval.

In this study, statistical analyses of EC data from the TP, YGP, and other regions of China were conducted to investigate ET variations across temporal scales, elevation, and LCT. Figure 2 depicts the variations in ET observed from EC at ARS (TP), QY station (Region I), ALS station (YGP), and QYZ station (Region II). The results show that daily ET exhibits seasonal variations. Specifically, daily ET ranges from –0.28 to 7.22 mm/day, with an average ET of 1.61±1.55 mm/day, and the 95% confidence intervals (CI) are [1.56, 1.66], from 2016 to 2022 at ARS (Figure 2a). At the QY station, daily ET varied from 0.001 to 6.25 mm/day, with a mean of 1.24±1.07 (CI=[1.18,1.31]) mm/day in 2018–2020 (Figure 2b). Although there is a small discrepancy in the daily ET observed at ARS and QY stations, with a difference of 0.37 mm/day, they exhibit different seasonal variations. The comparable average daily ET magnitudes are attributable to the fact that both monitoring stations are located in arid and semi-arid regions. There are significant differences in ET between ALS and QYZ EC stations (Figure 2c,d). At the ALS station, daily ET varied from 0.18 to 5.65 mm/day, with an average of 2.11±1.07 (CI=[2.06,2.14]) mm/day, exhibiting minor fluctuations over 2008–2013 (Figure 2c). In contrast, daily ET at the QYZ station varied from 0 to 6.52 mm/day, with an average of 1.88±1.34 (CI=[1.82,1.93]) mm/day, during 2003–2010 (Figure 2d). It is noted that the ET range at QYZ is larger than at ALS, but the mean ET is lower. In summary, there are marked regional differences in the daily ET observed by EC between the plateau and lowland study areas.

**Figure 3:**
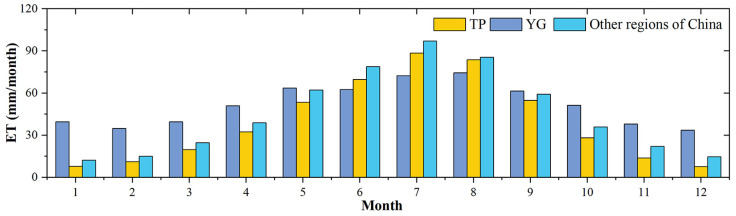
Monthly variations of ET in the YGP, TP, and other regions of China.

To further analyze differences in ET across regions, Figure 3 shows changes in the monthly average ET in TP, YGP, and other regions of China. The results show that the monthly average ET for the 16 EC stations on the TP exhibits a single-peak distribution, peaking in July at 87.04 mm/month and reaching a minimum in December at 7.91 mm/month, with an average of 38.91±29.15 (CI=[22.42,55.41]) mm/month. Although the monthly mean ET on the YGP also displays a single-peak pattern, this variation is relatively weak. The peak occurs in August at 74.40 mm/month, while the minimum occurs in December at 33.57 mm/month, with an average of 51.86±14.74 (CI=[43.51,60.20]) mm/month. Furthermore, high Ts results in higher ET on the YGP than in the TP. In the relatively lowland region of China, the monthly average ET exhibits a more significant cyclical fluctuation with a single-peak pattern than TP and YGP, with a minimum of 12.26 mm/month in January and a maximum of 97.05 mm/month in July, with an average of 45.51±30.06 (CI=[28.50,62.52]) mm/month.

**Figure 4:**
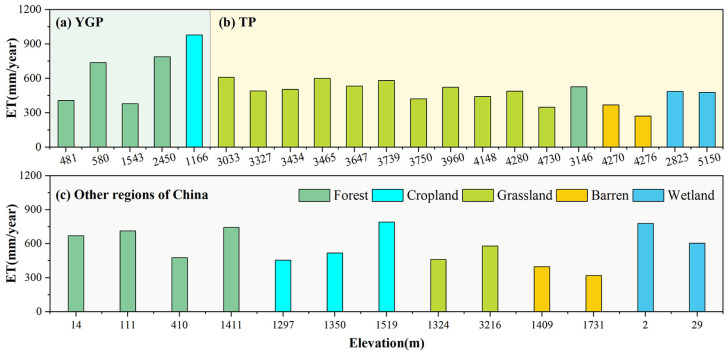
Average annual ET variations across different land cover types and elevations: (**a**) YGP, (**b**) TP, (**c**) other regions of China.

Given that ET varies considerably across different LCTs and elevations [23,68], Figure 4 shows the annual ET observed from EC at different LCTs and elevations. On YGP, ET in forests generally increases with altitude, except at the 1543 m station (Figure 4a). The lowest annual ET was recorded at the JFS station (1543 m) with an ET of 376.25 mm/year, and the highest at ALS (2450 m) with an ET of 788.72 mm/year. However, the annual ET at the GZPD station (1166 m) is significantly higher, at 978.85 mm/year, possibly attributable to irrigation practices. On the TP, annual ET at various grassland EC stations shows a decreasing trend with increasing altitude, a trend also observed at desert stations (Figure 4b). Notably, the ET values for wetlands at 2823 m (SGH) and 5150 m (TGL) are similar, with annual ET values of 486.63 mm/year and 478.81 mm/year, respectively. In other relatively lowland regions of China, the EC system records ET changes ranging from 318.88 to 790.56 mm/year, with significant variations across different elevations and LCTs (Figure 4c). Specifically, annual ET at forest EC stations generally increases with altitude, except at QYZ (111 m). The trend in ET at EC stations in farmland and grassland areas also indicates an increase with rising elevation. Collectively, these results indicate that ET varies significantly across different elevations and LCT.

### 4.2. Validation of ET Products Against EC Observations

**Figure 5:**
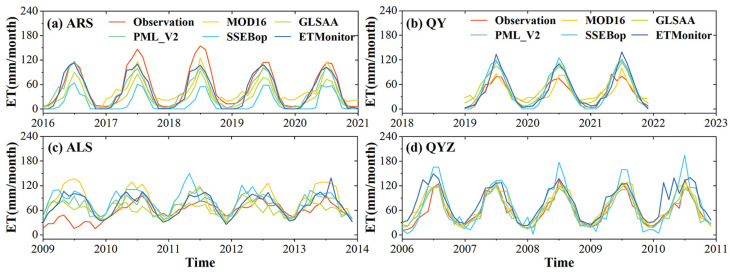
Comparison of ET observed by EC with five ET products in plateau and plain regions: (**a**) ARS at TP; (**b**) QY in Region I; (**c**) ALS at YGP; (**d**) QYZ in Region II.

Figure 5 presents a comparison of the temporal variations between the five ET products and the ET measurements at the TP, YGP, Regions I, and II. At the ARS station in TP, the five ET products exhibit consistent periodic variations with the observed ET but marked differences in ET magnitude (Figure 5a). Specifically, ETMonitor demonstrated the optimal performance, with an R^2^ of 0.92, an RMSE of 15.62 mm/month, and a bias of −8.90 mm/month. At the same time, the GLASS product exhibited the lowest accuracy, with an R^2^ of 0.89, an RMSE of 32.98 mm/month, and a bias of −24.45 mm/month. The overall accuracy of the five ET products at the QY EC station (in Region I) was superior to that observed at the ARS station (Figure 5b). The PML_V2 performed best, with a lower RMSE and bias of 15.76 and 7.60 mm/month, respectively. At the same time, the ETMonitor dataset yielded the poorest results, with an RMSE of 21.86 and a bias of 9.28 mm/month. At the ALS station in the YGP, the five ET products showed poor consistency with the observed ET. In contrast, the ET products at QYZ (in Region II) showed greater consistency with the observations than ALS, with all R^2^ values exceeding 0.71 (Figure 5c,d). In summary, these results indicate that the accuracy of the five products was lower at the ARS (TP) and ALS (YGP) stations compared to the GSQY and QYZ stations located in relatively flat areas.

**Figure 6:**
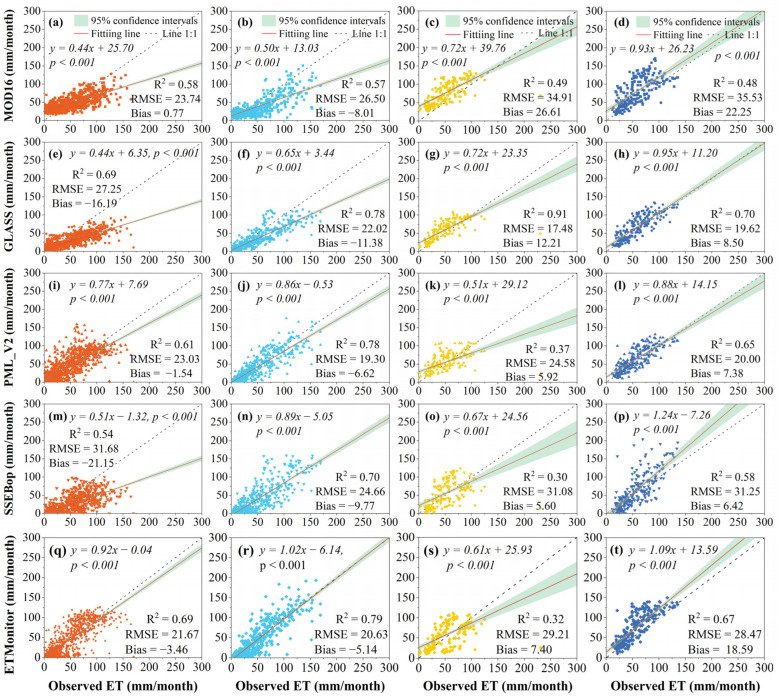
Comparison of ET products with observed ET: (**a**) MOD16 versus observed ET in the TP; (**b**) MOD16 versus observed ET in the Region I; (**c**) MOD16 versus observed ET in the YGP; (**d**) MOD16 versus observed ET in the Region II; (**e**–**h**) are the GLASS versus observed ET in TP, Region I, YGP, and Region II; (**i**–**l**) are the PML_V2 versus observed ET in TP, Region I, YGP, and Region II; (**m**–**p**) are the SSEBop versus observed ET in TP, Region I, YGP, and Region II; (**q**–**t**) are the ETMonitor versus observed ET in TP, Region I, YGP, and Region II.

**Figure 7:**
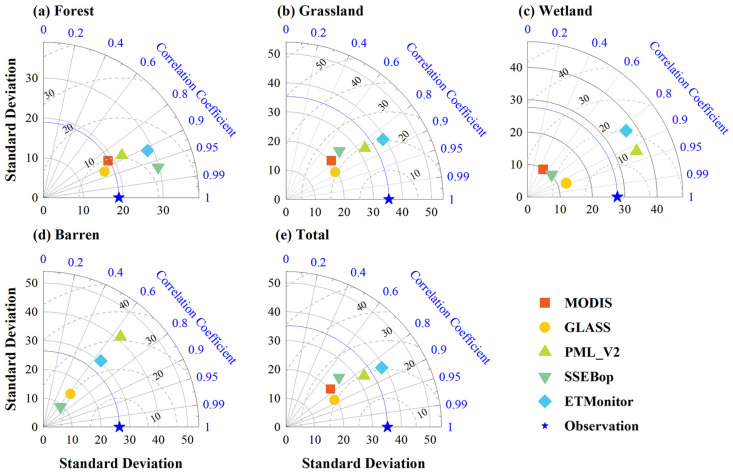
Taylor diagrams of five ET products against EC references aggregated across different LCTs in the TP.

**Figure 8:**
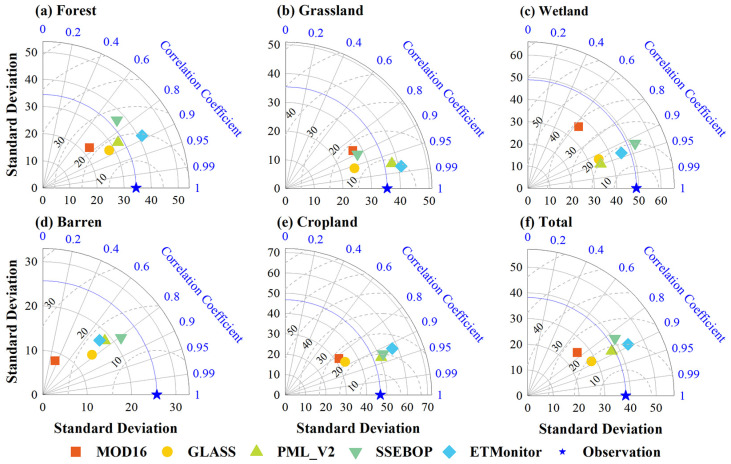
Taylor diagrams of five ET products against EC references aggregated across different LCTs in Region I.

To further quantify the accuracy of the five ET products across the TP, YGP, and Regions I and II, Figure 6 presents scatter plots comparing each ET product with EC observations. Figure 7 and Figure 8 present Taylor diagrams comparing ET from the five ET products with EC observations for various LCTs on the TP and Regions I. In addition, Table 3 summarizes the accuracy of ET products across various LCTs on YGP and Region II.

On the TP, significant variations in accuracy were observed among the five ET products (Figure 6a,e,i,m,q). The PML_V2 and ETMonitor demonstrated superior accuracy, achieving R^2^ of 0.61 and 0.69, RMSE of 23.03 and 21.67 mm/month, and biases of −1.54 and −3.46 mm/month, respectively (Figure 6i,q). Conversely, the SSEBop yielded the poorest accuracy, with an R^2^ of 0.54, an RMSE of 31.68 mm/month, and a bias of −21.15 mm/month (Figure 6m). As shown in Figure 7, there are substantial discrepancies among ET products across different LCT. For forests, all ET products show high consistency with observed ET, especially for GLASS, with all R exceeding 0.8, RMSE below 20 mm/month, and standard deviations (STD) below 30 mm/month (Figure 7a). For grassland and wetland, PML_V2 displayed the highest agreement with observed ET, achieving an R above 0.84, an RMSE below 20 mm/month, and an STD below 35 mm/month (Figure 7b,c). At the barren, ETMonitor exhibited the highest performance (Figure 7d). Across all LCTs, the PML_V2 and ETMonitor products outperformed other ET products (Figure 6a,e,i,m,q and Figure 7e). In summary, PML_V2 is recommended for grassland/wetland ecosystems, while ETMonitor is recommended for barren areas on the TP.

In Region I, the performance of most ET products was superior to that observed in the TP (Figure 6b,f,j,n,r). Specifically, the PML_V2 product continued to perform best, with an R^2^ of 0.78, an RMSE of 19.30 mm/month, and a bias of −6.62 mm/month (Figure 6j,i). ETMonitor also demonstrated high consistency with the observed ET, with an R^2^ of 0.79 and RMSE and bias of 20.63 and −5.14 mm/month, respectively (Figure 6r). GLASS and SSEBop displayed moderate performance, although GLASS achieved a relatively higher R^2^. Conversely, the MOD16 product exhibited the lowest performance in this region, with an R^2^ of 0.55, an RMSE of 26.50 mm/month, and significant underestimation (bias of −8.01 mm/month) (Figure 6b). Significant differences also exist in the performance of the five ET products across various ecosystems within Region I (Figure 8). In forests, GLASS exhibited the highest performance, with an R of 0.87, an RMSE of less than 20 mm/month, and an STD of 28.15 mm/month; conversely, SSEBop demonstrated the lowest accuracy (Figure 8a). In grasslands, PML_V2 and ETMonitor showed strong agreement with the observed ET, with R consistently greater than 0.87 and RMSEs below 10 mm/month. Furthermore, the STDs of both products were comparable to those observed for the ET. MOD16 exhibited the least accuracy in grasslands (Figure 8b). Regarding wetlands, a significant discrepancy was observed between the five ET products and observed ET, with STDs ranging from 30 to 60 mm/month and RMSEs below 25 mm/month (Figure 8c). Despite the general variability, ETMonitor and SSEBop showed good agreement with the observed ET. However, MOD16 continued to perform the worst in wetlands (Figure 8c). In barren lands, SSEBop showed the best agreement with the observed ET, with an R of 0.75, an STD of 21.80 mm/month, and an RMSE of less than 20 mm/month, whereas the MOD16 still exhibited the poorest performance (Figure 8d). Furthermore, PML_V2 and SSEBop showed the best agreement with observations in croplands, with an R of 0.88, an RMSE below 20 mm/month, and an STD closest to the observed ET (Figure 8e). Across all LCTs, PML_V2 demonstrated the best agreement with ET observations among the five products (Figure 6b,f,j,n,r and Figure 8f). Therefore, the GLASS product is recommended for forest ecosystems. For grassland and cropland ecosystems, the PML_V2 is advised. The ETMonitor is suitable for wetland areas, and the SSEBop model is recommended for barren regions in Region I.

On the YGP, in contrast to the superior performance of GLASS, the other products exhibited relatively lower accuracy (Figure 6c,g,k,o,s). Specifically, the GLASS demonstrated relatively high accuracy, with an R^2^ of 0.91, an RMSE of 17.48 mm/month, and a bias of 12.21 mm/month (Figure 6g). The MOD16 still exhibited the lowest performance, with an R^2^ of 0.49, an RMSE of 34.91 mm/month, and a bias of 26.61 mm/month (Figure 6c). Furthermore, the PML_V2, SSEBop, and ETMonitor products showed relatively low accuracy, with R^2^ below 0.37, RMSE ranging from 24.58 to 31.08 mm/month, and biases between 5.60 and 7.40 mm/month (Figure 6k,o,s). There is considerable variation in the accuracy across forest and cropland ecosystems in YGP (Table 3). For forests, the accuracy of the five ET products was poor, with R^2^ remaining below 0.53. Specifically, the GLASS exhibited relatively higher accuracy, with an R^2^ of 0.53, an RMSE of 23.66 mm/month, and a bias of 9.38 mm/month. The MOD16 had the poorest accuracy, with an RMSE of 35.13 mm/month and a bias of 25.16 mm/month. In cropland ecosystems, all five ET products demonstrated high accuracy, with R^2^ consistently exceeding 0.75. Specifically, the GLASS and PML_V2 remained the best performers, with R2 of 0.91 and 0.78, RMSE of 17.48 and 11.19 mm/month, and bias of 12.21 and 7.21 mm/month. The MOD16 showed the poorest agreement with observed ET, with an R^2^ of 0.75, an RMSE of 34.29 mm/month, and a bias of 30.81 mm/month. In YGP, the GLASS product is recommended for forest ecosystems, while the PML_V2 is advised for cropland ecosystems.

In Region II, most ET products showed better agreement with observed ET than those in the YGP, except for MOD16 (Figure 6d,h,l,p,t). For instance, the PML_V2 and GLASS exhibit high accuracy, with R^2^ of 0.70 and 0.65, RMSE of 19.62 and 20.00 mm/month, and biases of 8.5 and 7.38 mm/month, respectively (Figure 6h,l). Conversely, MOD16 continued to exhibit the lowest accuracy with an R^2^ of 0.48, an RMSE of 35.53 mm/month, and significant overestimation (bias = 22.25 mm/month). In addition, both GLASS and PML_V2 demonstrated good accuracy on the forest, with R^2^ of 0.75 and 0.68, RMSEs of 19.76 and 19.22 mm/month, and biases of 11.27 and 8.37 mm/month, respectively (Table 3). MOD16 also performed the worst in this region, with an RMSE of 36.77 mm/month and a bias of 23.95 mm/month, respectively, although its accuracy was superior to that in the forests. Regarding wetlands, the GLASS exhibited the highest accuracy, with an R^2^ of 0.75, an RMSE of 18.73 mm/month, and a bias of 11.27 mm/month; conversely, SSEBop performed the worst, with an R^2^ of 0.43, an RMSE and a bias of 55.56 and 20.38 mm/month, respectively. In Region II, GLASS performs best and is recommended for forest and wetland ecosystems.

In summary, the accuracy of the five ET products is higher in relatively lowland flat regions than in plateau regions. However, marked differences exist in the performance of the five ET products across different LCTs in different regions. On the TP, PML_V2 demonstrated superior performance in grasslands and wetlands, whereas GLASS performed best in forests, and ETMonitor excelled in barren land. In Region I, PML_V2 had high accuracy in forests, grasslands, and croplands; ETMonitor showed better accuracy in wetlands, and SSEBop performed well in barren areas. In the YGP, GLASS performed well in forests, while PML_V2 performed well in cropland. In Region II, GLASS exhibited the best performance in forests and wetlands. The SSEBop exhibited moderate accuracy across most study regions, performing better in relatively lowland regions. Although MOD16 had lower accuracy across all regions, it performed adequately in specific LCTs (e.g., forests on the TP and croplands in the YGP) and can still be used for regional ET estimation.

### 4.3. Spatiotemporal Differences in ET Between the Plateau and the Plain

**Figure 9:**
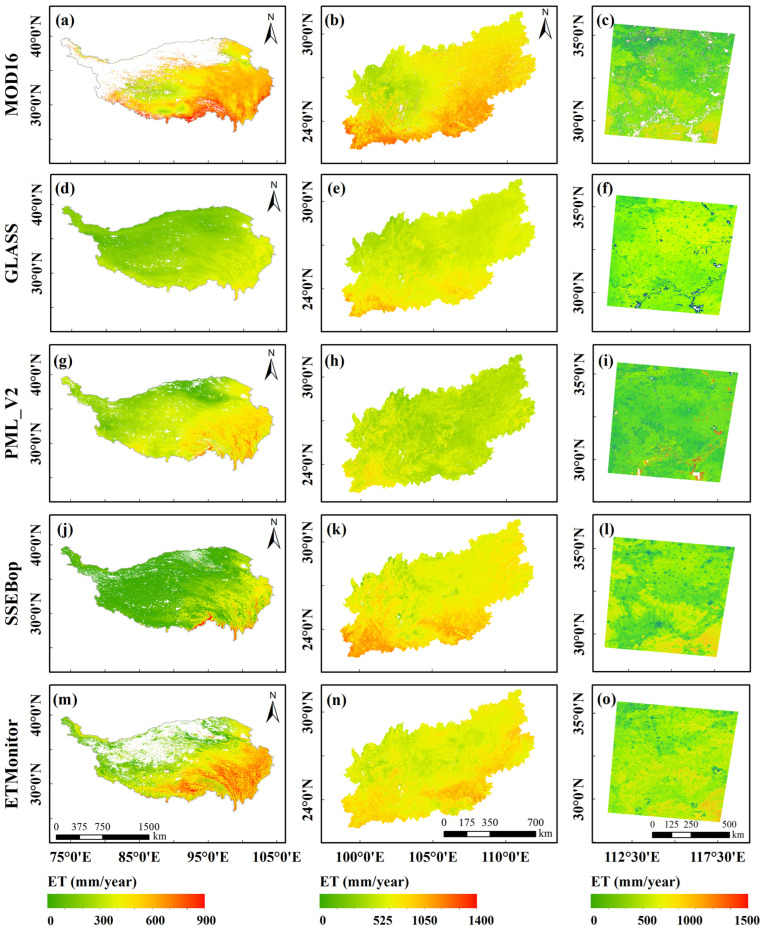
Spatial distributions of ET for five products in 2020 across different areas: (**a**–**c**) are MOD16 over TP, YG, and HYP, respectively; (**d**–**f**) are the GLASS over the TP, YG, and HYP, respectively; (**g**–**i**) are the PML_V2 over the TP, YG, and HYP, respectively; (**j**–**l**) are the SSEBop over the TP, YG, and HYP, respectively; (**m**–**o**) are the ETMonitor over the TP, YG, and HYP, respectively.

Figure 9 illustrates the spatial distribution of annual ET from these five ET products in 2020 across the TP, the YGP, and HYP. In the TP, although all five ET products exhibit a gradual decrease from southeast to northwest, there are significant differences in spatial distribution with topographical variations (Figure 9a,d,g,j,m). Specifically, ETMonitor and PML_V2 exhibit distinct variations with terrain change, particularly in the southwestern part of the TP. The SSEBop exhibits a moderately pronounced spatial distribution with topographical changes (Figure 9j). MOD16 exhibits a spatial distribution pattern characterized by abnormally high ET in the southwest, significant regional variations that do not reflect topographical features, and distinct noisy pixels with high ET along the edges of water bodies (Figure 9a). In contrast, GLASS shows weaker variation in response to topographic changes (Figure 9b). It is noteworthy that all ET maps exhibit data gaps on the TP. MOD16 has extensive data gaps, particularly in the northwestern part of the TP; the ETMonitor also shows similar gaps (Figure 9m). The PML_V2 and SSEBop have small gaps in the northwest, whereas GLASS demonstrates the best spatial continuity.

On the YGP, the spatial continuity of the five ET products is better than that in the TP (Figure 9b,e,h,k,n), and each product exhibits a spatial pattern with higher ET in the south or southwest. Specifically, MOD16 shows higher ET in the south and southwest, whereas in the northwest, ET is lower and shows little variation with terrain (Figure 9b). Analogously, GLASS shows high ET in the southwestern region and low ET in other areas lacking distinct topographic distribution patterns (Figure 9e). As shown in Figure 9h, although the ET values of PML_V2 are lower than those of other products, there is a distinct spatial distribution that corresponds to terrain variations. SSEBop and ETMonitor show high ETs in the southwestern and southeastern regions, exhibiting a weak topography-dependent distribution (Figure 9k,n).

In the HYP, the five ET products exhibit similar spatial distributions (Figure 9c,f,i,l,o). Specifically, MOD16 can capture features associated with land cover changes but fails to represent water bodies, resulting in significant errors in their vicinity (Figure 9c). GLASS exhibits a more continuous spatial distribution with minimal fluctuations, characterized by higher ET in the north and lower values in the south (Figure 9f). Although the overall PML_V2 is relatively low, it effectively reflects changes in land cover and shows distinct high ET over water bodies (Figure 9i). SSEBop and ETMonitor (Figure 9l,o) show clearer variations in topography and LCT, with higher ET in the southern hilly areas and lower ET in the northern plains.

In summary, significant differences exist in the spatial continuity and spatial patterns among the five ET products on plateaus and plains. PML_V2, SSEBop, and ETMonitor display high terrain-dependent variation across all plateaus. MOD16 exhibits significant spatial variations in TP and YGP that do not correspond to the terrain distribution but show good spatial continuity in relatively flat areas. Although GLASS demonstrates good spatial continuity across all regions, it lacks terrain-dependent variation. In the plain region (HYR), the spatial continuity and patterns of the five ET products are similar.

**Figure 10:**
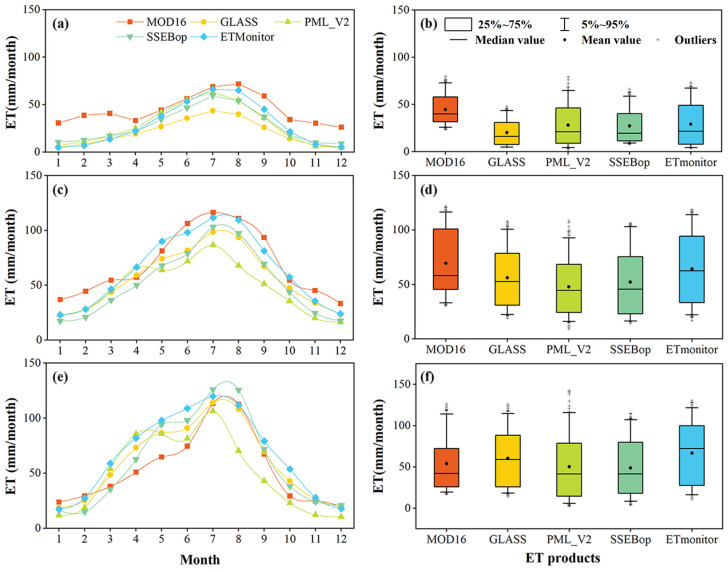
Seasonal cycles and monthly boxplots of ET for five products from 2003 to 2020: (**a**) seasonal cycles in the TP; (**b**) monthly ET boxplots in the TP; (**c**,**d**) in YGP; (**e**,**f**) in HRP.

Figure 10 displays the seasonal fluctuations in the monthly mean values of the five ET products across the TP, YG, and HYP from 2003 to 2020, along with box plots illustrating the monthly ET distribution. Overall, all ET products exhibited a single-peak pattern, with peaks concentrated in July and August and minimum ET occurring in December and January (Figure 10a,c,e). However, there were larger discrepancies in the magnitudes of monthly ET among the three regions (Figure 10b,d,f).

In the TP, the overall ET was the lowest, with monthly ET ranging from 4.61 ± 1.56 (PML, December, CI = [4.585, 5.168]) to 71.76 ± 4.46 (MOD16, August, CI = [4.585, 5.168]) mm/month. Specifically, MOD16 was generally higher than other products, particularly during the non-growing season (January–April and October–December) (Figure 10a). PML_V2, SSEBop, and ETMonitor demonstrated good consistency across all months. Conversely, GLASS was generally lower throughout the year, particularly during the growing season. During the study period, the monthly ET box plots for the five ET products (Figure 10b) indicate that MOD16 was significantly higher than the other ET products, with a 25–75% interquartile range (IQR) of 31.74–57.88 mm/month, and median and mean values of 40.06 and 44.6 mm/month, respectively. GLASS exhibited a lower median and mean ET of 16.27 and 20.39 mm/month, respectively, and a narrower range (7.69–30.87 mm/month). PML_V2, SSEBop, and ETMonitor exhibited similar medians (20.98, 19.59, and 21.65 mm/month) and means (28.08, 27.48, and 29.21 mm/month). The numerical ranges of PML_V2 and ETMonitor are consistent and larger than those of the other products, at 8.90–46.41 and 7.81–49.01 mm/month, respectively, reflecting a greater ability to capture ET heterogeneity in complex terrain. Furthermore, Figure 6 and Figure 9 demonstrate that both products accurately represent the spatiotemporal distribution characteristics of the TP, both in numerical values and spatial distribution.

In the YGP (Figure 10c), the five ET products demonstrated high consistency, and the ET was higher than that of the TP, with monthly ET ranging from 16.48 ± 3.56 (PML, December, CI = [14.85, 18.14]) to 111.63 ± 3.63 (MOD16, July, CI = [114.65, 118.01]) mm/month. Specifically, ETMonitor and MOD16 exhibited the highest peaks during the growing season (June–August), while PML_V2′s ET were generally lower across all seasons. Conversely, SSEBop and GLASS demonstrated good consistency in all periods. During the study period, MOD16 and ETMonitor (Figure 10d) exhibited wider variability in the YGP, with the largest dispersion occurring within the 25–75% IQR (45.42–100.96 mm/month), and higher medians and means (58.13 and 69.58 mm/month, and 62.51 and 64.21 mm/month, respectively), reflecting their greater variability and generally higher magnitude in this region. In contrast, the medians (52.66, 44.53, and 45.73 mm/month) and means of GLASS, PML_V2, and SSEBop were relatively close, and the 25–75% IQRs showed minimal differences, indicating good consistency among these products.

In the HYP, the ET from the five ET products was higher than that in the two plateau regions, with a monthly ET range of 10.37 ± 3.97 (PML, December, CI = [8.54, 12.21]) to 126.01 ± 66.85 (SSEBop, July, CI = [122.85, 129.17]) mm/month and the greatest seasonal variation (Figure 10e). Specifically, the ET from the five ET products showed greater convergence in July and August, whereas significant discrepancies were observed from January to April. From January to April, MOD16 was notably lower, while PML_V2 was also significantly lower from August to October. Throughout the study period (Figure 10f), ETMonitor displayed the highest median of ET (72.29 mm/month) and the widest distribution range (25–75% IQR of 27.51–100.00 mm/month), whereas GLASS exhibited a moderate distribution, with a median and mean of 58.86 and 60.31 mm/month, respectively, and a 25–75% IQR of 25.82–88.21 mm/month. For PML_V2 and SSEBop, the median (41.68 and 41.67 mm/month) and mean (50.23 and 48.92 mm/month) were relatively close, as were their 25–75% IQRs, indicating consistent variability in these data for PML_V2 and SSEBop in this region. In contrast, the median for MOD16 was lower (Figure 10e), consistent with its tendency to yield lower ET during the non-growing season, suggesting an underestimation in the plain region.

**Figure 11:**
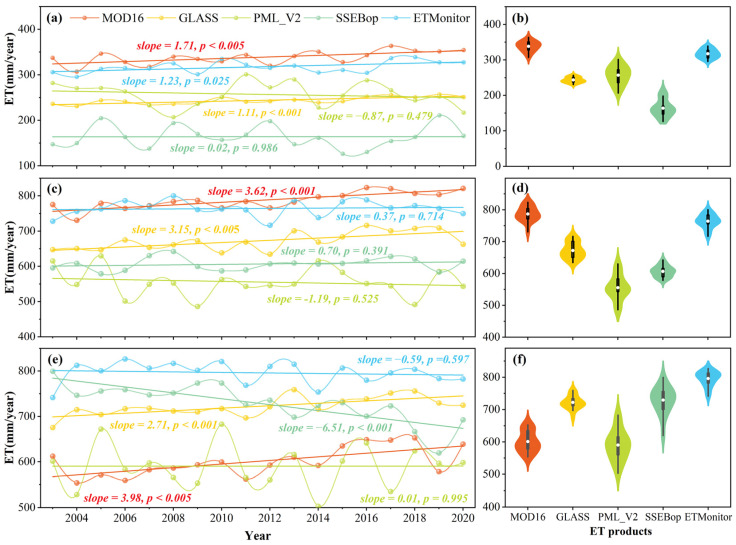
Interannual variations and yearly boxplots of ET for five products from 2003 to 2020: (**a**) interannual variations in TP; (**b**) yearly ET boxplots in TP; (**c**,**d**) in the YGP; (**e**,**f**) HRP. The horizontal width of each violin curve denotes the relative sample density of ET at the corresponding vertical position.

Figure 11 illustrates the temporal trends in annual ET across the five ET products, along with violin plots for TP, YGP, and HYP. In the TP, as shown in Figure 11a, MOD16, GLASS, and ETMonitor exhibited consistent, significant increasing trends from 2003 to 2020 (slopes of 1.71, 1.23, and 1.11 mm/year, respectively, *p* < 0.05). In contrast, PML_V2 showed a slightly decreasing trend with a slope of −0.87 mm/year (*p* = 0.479), while no statistically significant monotonic trends were detected for SSEBop (slope of 0.02 mm/year, *p* = 0.986). With respect to the statistical distribution of the five products (Figure 11b), MOD16 and ETMonitor displayed higher annual mean values (338.2 and 317.37 mm/year, respectively) and a more concentrated distribution. Meanwhile, GLASS and PML_V2 showed similar ET magnitudes (243.60 and 257.04 mm/year, respectively); PML_V2 exhibited a wider range of ETs. Conversely, SSEBop had the lowest ET (163.95 mm/year) yet demonstrated high variability.

In the YGP, the overall trend in annual mean ET is similar to that observed in the TP, albeit with greater variability (Figure 11c,d). Both MOD16 and GLASS exhibit significant upward trends, with slopes of 3.62 (*p* < 0.001) and 3.15 (*p* < 0.01), respectively. Conversely, ETMonitor and SSEBop display slight increasing trends with slopes of 0.37 and 0.70, respectively, which were not statistically significant. PML_V2 fluctuates considerably over time and exhibits a slight downward trend, although this is not statistically significant. As shown in Figure 11d, the mean values for ETMonitor and MOD16 are relatively high with 764.22 and 786.93 mm/year, respectively. The mean values for PML_V2, GLSS, and SSEBop are fairly similar. The PML_V2 data exhibit a wide range of variation, whereas the SSEBop data are more concentrated.

In the HYP, the annual ET also showed distinct temporal trends, but the magnitude of ET was greater than that observed in the plateau regions (Figure 11e,f). As shown in Figure 11e, MOD16 and GLASS exhibited clear increasing trends, with slopes of 2.71 (*p* < 0.001) and 3.98 (*p* < 0.001), respectively. In contrast, SSEBop exhibited a highly significant downward trend with a slope of −6.51 (*p* < 0.001). ETMonitor displayed a slight downward trend but lacked statistical significance. PML_V2 fluctuated significantly over time but showed no clear trend and was not statistically significant. The annual mean ET for MOD16 and PML_V2 were similar, at 600.87 and 590.33 mm/year, respectively (Figure 11f). Similarly, the mean ET for GLASS and SSEBop were comparable, at 722.04 and 729.08 mm/year, respectively. It is worth noting that the data distributions for PML_V2 and SSEBop were wider, whereas those for GLASS were more concentrated.

In conclusion, the five ET products show significant differences in temporal changes between the plateau and plain regions. MOD16 overestimated ET on the plateau but underestimated it on the plains, demonstrating a statistically significant increasing trend in all regions. ETMonitor consistently yielded higher ET estimates across all regions, exhibiting an increasing trend in the plateau and a non-significant decreasing trend in the plain. GLASS exhibited an upward trend across all three regions. SSEBop exhibited greater fluctuations in TP and plains than YGP. PML_V2 showed broad distribution across all three regions and lacked statistically significant monotonic trends, reflecting substantial response variability to changes in terrain and ecosystems.

### 4.4. Driving Factors of ET Products Between the Plateau and the Plain

**Figure 12:**
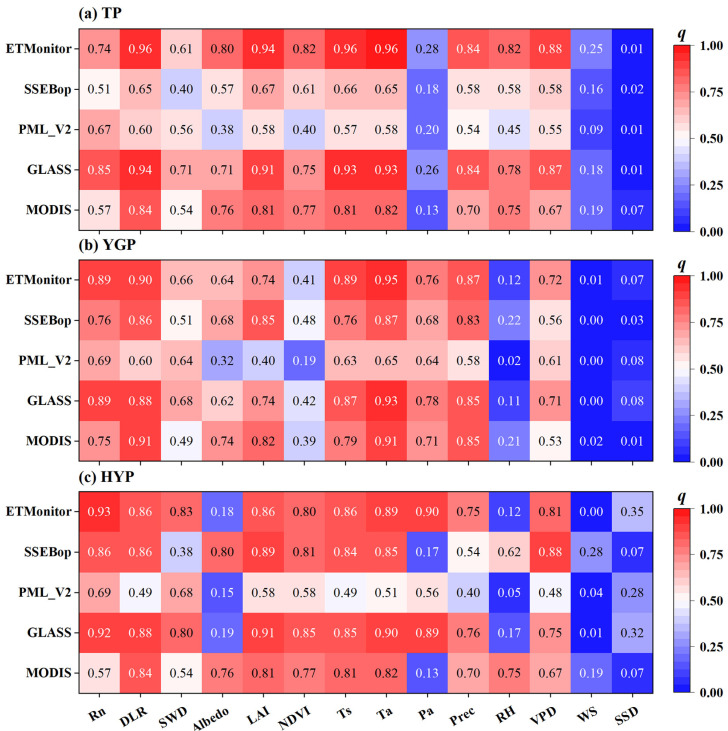
Heatmap of the drivers for five ET products across three regions: (**a**) TP; (**b**) YGP; (**c**) HYP.

Figure 12 presents a heatmap of the drivers for five ET products across three regions. The results indicate significant differences in the primary driving factors for each product across the different regions. On the TP (Figure 12a), the primary driving factor for MOD16, GLASS, and ETMonitor was DLR, with q-values of 0.84, 0.94, and 0.96, respectively. Ta and Ts were identified as secondary critical factors for the aforementioned three ET products, with q-statistics exceeding 0.80. Given the close correlation between Ta and DLR $\left(DLR=\varepsilon_{a}\sigma T_{a}^{4}\right)$ [67], this suggests a significant influence of heat-related factors on, ET processes in high-altitude cold regions. In contrast, the primary driving factor for PML_V2 was Rn (q = 0.67), whereas that for SSEBop was LAI (q = 0.96). Other factors, such as NDVI, Prec, albedo, and VPD, also exhibited a strong impact on ET over the TP, with q-values ranging from 0.54 to 0.88. This phenomenon potentially reflects the regulatory roles of vegetation growth, water supply, and surface properties on ET.

On the YGP (Figure 12b), Ta remains the key driving factor, with q-statistics exceeding 0.86 for most products. Specifically, the primary driving factors for MOD16 are DLR and Ta, with q-values of 0.91 for both. For GLASS, SSEBop, and ETMonitor, the primary driving factor is Ta, with q-values of 0.93, 0.87, and 0.95, respectively. PML_V2 continues to be primarily controlled by Rn, with a q-value of 0.69. However, compared with the TP, the explanatory power of Prec, Pa, and RH is greater for the YGP, reflecting the significant regulatory role of moisture conditions in YGP for ET. The low sensitivities of RH, WS, and SSD suggest that the spatial pattern of ET is primarily determined by a combination of thermal and moisture conditions rather than by a single meteorological factor.

For the plain region (Figure 12c), MOD16 still has DLR as its primary driving factor (q = 0.84); GLASS and ETMonitor shift to Rn as the dominant factor, with q-values of 0.92 and 0.93, respectively. PML_V2′s primary driving factor remains Rn (q = 0.69); SSEBop is driven by LAI, with a q-value of 0.89 (Figure 12c). Notably, compared with the plateau region, the q-value for Rn (q ≈ 0.57–0.93) is significantly higher, indicating a heightened sensitivity of ET to Rn in the plains. The explanatory power of vegetation factors (LAI and NDVI) in this region is significantly higher (q ≈ 0.49–0.91), reflecting the strong influence of vegetation phenology on ET in these agricultural ecosystems. Concurrently, the driving power of VPD and RH is also significantly higher (q ≈ 0.54–0.88), reflecting the significant role of atmospheric humidity conditions in the plains in determining ET.

In summary, MOD16, ETMonitor, and GLASS use radiation or temperature as their primary driving factors, whereas PML_V2 uses net Rn as its primary driving factor across the three regions. SSEBop has LAI as its main driving factor in the TP and HYP, and Ta as the main driving factor in the YGP. There are marked regional variations in the dominant driving factors: on the TP, thermal radiation factors (Ta, Ts, DLR) are predominant; the YGP is characterized by a combination of thermal and moisture factors, whereas the HYP exhibits a pattern of synergistic interaction between thermal, moisture, and vegetation factors.

## 5. Discussion

### 5.1. Uncertainties in ET Products Evaluation

#### 5.1.1. Uncertainties from EC Measurements

(1) Uncertainties arising from gap-filling. In general, monthly ET was calculated by aggregating EC observations obtained from hourly or half-hourly records. Missing and invalid data inevitably arise from instrument power outages, system errors, a lack of maintenance, and adverse weather conditions (e.g., heavy rain). For example, data gaps typically account for 17–50% of the half-hourly or hourly ET observations at FLUXNET sites [69,70]. In addition, aggregating the ET observed by EC from half-hourly or hourly to monthly scales may introduce errors, especially when there are significant data gaps. Jiang et al. [69] reported that the accuracy of ET gap-filling varies significantly with the employed method across different ecological vegetation zones, with RMSE values ranging from 18.8 to 95.9 W/m^2^ for daily gap-filling. In this study, the EC data used to validate the five ET products consisted of monthly ET data obtained directly from sources such as ChinaFLUX and HiWATER, along with ITPCAS data that underwent imputation, which significantly mitigated errors from gap-filling and interpolation. However, owing to substantial variability among gap-filling methods, the monthly ET derived from the three EC datasets in this study inevitably introduces uncertainty into the validation process.

(2) Uncertainties from energy-balance non-closure. Since data for key energy-balance components (i.e., net radiation, sensible heat flux, and soil heat flux) were missing at some EC sites, energy-balance closure corrections were not applied to the validation of the five ET products conducted with EC data in this study. Numerous studies have demonstrated that the magnitude of energy imbalance at the EC flux tower ranges from 10% to 30% [71,72,73]. Energy imbalances at EC stations typically result in an underestimation of LE or an overestimation of available energy. Failure to correct energy-balance closure corrections may not only lead to biases in the validation results of ET products but also significantly reduce the accuracy of spatiotemporal analysis of ET products in plateau and plain regions. In future research, we will collect data from EC observation stations for which energy closure rates are available and apply energy-balance corrections to the observed LE to improve the reliability of the validation results.

(3) Uncertainties from footprint mismatch. In this study, we extracted ET values from the five products using a 3 × 3-pixel window centered on each EC station and compared the window-mean ET with in situ EC observations. Chu et al. [68] indicated that the footprint observed by EC ranges from 250 m to 3000 m. The spatial scale discrepancy between ground measurements and satellite observations may introduce significant uncertainties, particularly on heterogeneous surfaces [74,75]. Xie et al. [3] report that, when EC measurements are used to evaluate ET products at the point scale, uncertainties arising from scaling effects vary from 5% to 25%. Previous studies have shown that ET products yielded very similar results on homogeneous surfaces, but their differences increased by as much as 10% on heterogeneous surfaces [37,76]. Given the complex terrain, the uncertainty arising from this mismatch may be even greater in the TP and YGP. In future research, we intend to utilize higher-resolution RS ET data, large-aperture scintillometers, or a weighted EC footprint validation approach to mitigate validation biases caused by scale mismatches.

#### 5.1.2. Uncertainties from RS-Based Data

There are variations in the temporal resolution of different products, such as the 8-day resolution of MOD16 and GLASS compared to the 1-day resolution of PML_V2, which may introduce some uncertainty when aggregating data to a monthly ET. Additionally, gaps in ET maps that occur periodically due to factors such as cloud contamination reduce the number of valid pixels available for monthly-scale aggregation. Consequently, aggregating ET to monthly-scale products introduces additional uncertainties, particularly in plateau regions, which then propagate to the final validation results.

The spatial resolution varies across ET products; for instance, MOD16 and PML_V2 have a resolution of 500 m, whereas GLASS, SSEBop, and ETMonitor have a resolution of 1000 m. To standardize the spatial resolution, we employed bilinear interpolation to resample the 1000 m products to 500 m, a process that may also introduce uncertainty into the validation. In plateau regions, complex terrain and steep topographic gradients within satellite pixels can compromise resampling accuracy, thereby affecting the validation results.

#### 5.1.3. Uncertainty in the Detection of Driving Factors

This study employed Geodetector to investigate the driving factors of ET across different regions. However, as Geodetector is primarily based on stratified heterogeneity analysis, it may have limitations in fully capturing complex nonlinear relationships among variables (e.g., the linear correlation between net radiation and radiation components versus the nonlinear relationship with temperature) [77]. Future studies will attempt to integrate Random Forest models to better analyze these nonlinear effects.

Uncertainties also arise from the driving factor data themselves. Mismatches in spatiotemporal resolution and origins among multisource data (e.g., GLASS and CMFD) may compromise the accuracy of factor identification. Furthermore, the bilinear interpolation used for downscaling meteorological forcing data relies solely on neighboring grid cell values, ignoring spatial heterogeneity and geographic supports. Consequently, geographically weighted regression is expected to provide more accurate interpolation.

Additionally, vegetation parameterization presents limitations. Using monthly averages aggregated from 8-day or 16-day data (e.g., MOD13A2 and GLASS) may fail to capture rapid vegetation changes and seasonal phenology variations. Moreover, the lack of distinction among different LCTs may introduce bias into the detection results.

### 5.2. Potential Cause of the Difference in ET Between Plateaus and Plains

#### 5.2.1. Difference in the Temporal Variability of ET Across Different Regions

There are distinct ET peaks in the two plateaus, with the ET peak in the YGP occurring 1 month later than in the TP but having a higher monthly average (see Figure 3). This phenomenon may be attributed to the fact that the YGP lies within the low-latitude plateau monsoon and subtropical monsoon climate zones, where rainfall is abundant year-round, and high forest cover provides a substantial water supply for ET.

#### 5.2.2. Difference in Accuracy of ET Products and Improvements in Their Algorithm

This study found that the accuracy of the five ET products is higher in relatively low-lying flat regions than in topographically complex plateau regions. This observation is consistent with the findings of Liu et al. [23], who reported that most ET products perform better at lower elevations. Furthermore, the accuracy of the five ET products varies significantly across different LCT. The estimates derived from PM-based ET models (PML_V2 and ETMonitor) exhibit high accuracy and display distinct topographic distribution patterns on the TP, a finding consistent with the conclusions of Zheng et al. [47]. Specifically, PML_V2 demonstrates high accuracy, particularly within grassland and wetland ecosystems. This superior performance may be attributed to the fact that PML_V2 calculates ET by partitioning it into three distinct components: soil evaporation ($E_{s}$), plant canopy transpiration ($E_{t}$), evaporation of precipitation intercepted by vegetation ($E_{i}$). In particular, for $E_{t}$ estimation, PML_V2 employs a water–carbon coupled canopy conductance model to derive canopy transpiration and GPP [56]; additionally, the model parameters are calibrated using in-situ data [78], which facilitates the acquisition of more accurate estimates in regions with abundant vegetation. Given that canopy conductance governs both transpiration and photosynthesis, PML_V2 yields highly accurate estimates for vegetation-dominated ecosystems. Furthermore, owing to its high spatial resolution (500 m), this product captures more detailed topographic variations compared to other products.

ETMonitor also exhibits high accuracy, particularly in barren ecosystems, whereas grassland and wetland ecosystems demonstrate relatively high accuracy for TP. This performance is primarily attributed to the model’s comprehensive consideration of the contribution of soil, vegetation, water bodies ($E_{w}$), and snow and ice ($E_{ss}$) when estimating ET ($ET=E_{t}+E_{s}+E_{i}+E_{w}+E_{ss}$) [57]. In addition, ETMonitor uses high-resolution soil moisture data to refine soil parameterizations for estimating soil evaporation. Consequently, ETMonitor achieves higher accuracy in barren regions. Regarding topographic correction, a digital elevation model (DEM) is used to downscale the soil moisture data; therefore, ETMonitor reflects distinct topographic variations in spatial distribution on the plateau.

MOD16 exhibited poor overall performance across all regions; however, it demonstrated adequate accuracy in specific LCTs (e.g., forests on the TP and croplands in the YGP). Similar findings have been reported in previous studies [1,47,79]. This limitation arises because the MODIS algorithm employs a modified PM approach to estimate daytime ET only, neglecting ground heat flux, $E_{i}$, $E_{s}$, and $E_{w}$, and nighttime ET [19,57]. Furthermore, the model input parameters do not account for topographic effects (such as Ts, Ta, VPD, and Rn). Consequently, the accuracy of MOD16 is relatively low in mountainous areas. However, the MODIS product, derived from polar-orbiting satellites, provides unparalleled, temporally and spatially continuous information regarding vegetation and surface energy, facilitating its use for regional ET estimates [80].

To enhance the accuracy of PM-based ET models in mountainous regions, it is imperative to comprehensively incorporate soil moisture and vegetation phenology data and to use topographic information to calibrate key input parameters, such as Ts, Rn, and soil moisture content. For example, Di et al. [79] integrated two soil moisture layers into the MOD16 algorithm, thereby significantly improving its performance. Yuan et al. [81] enhanced a PM-based model by developing an algorithm to estimate ET that incorporates soil moisture data and optimizes canopy transpiration across the TP. Similarly, Shang et al. [82] estimated canopy conductance using machine learning methods and coupled it with PM-type models to improve ET estimation accuracy in the TP.

GLASS achieved higher accuracy in forest areas across the TP and YGP. Xie et al. [3] and Wang et al. [83] also reported that GLASS exhibited superior accuracy for forests at regional and global scales in China. The underlying reasons for this phenomenon may be attributed to the GLASS ET algorithm’s multi-algorithm integration strategy, which uses the BMA method and EC data from 240 flux stations worldwide to integrate five PM-based algorithms [22,84]. In more detail, GLASS utilized extensive EC observations from various forest types, which may contribute to high accuracy in forested regions [22]. Consequently, GLASS generally improves the accuracy of ET estimates compared to individual algorithms, optimizes fused algorithms, and refines input parameters by considering terrain effects.

The SSEBop model exhibits moderate performance across all regions, with degraded accuracy over the plateaus. The SSEBop model estimates using the formula $ET={ET}_{f}\times k\times {ET}_{r}$, which is the product of an ET fraction (${ET}_{f}$) generated from Ts, coefficient k (1.25), and reference ET ${(ET}_{r})$ [20]. The most important assumption of the model is that the surface energy balance process is primarily driven by Rn, and a decline in ET due to water stress and other factors can be quantified by differences in Ts [85]. Due to the complex topography of plateau regions, there are significant variations in Rn, Ts, and Ta, and the neglect of LCT variations renders the SSEBop model’s assumptions unsuitable for mountainous areas. Furthermore, its fixed parameters, specifically the constant coefficient k used to scale the grass reference ET and aerodynamic resistance, may also be inaccurate under the heterogeneous conditions found in mountainous regions [86]. These factors collectively contribute to the reduced accuracy of SSEBop in mountainous areas. Given that radiation and temperature are the key input parameters and primary driving factors for SSEBop across all three study areas (see Figure 12), SSEBop can be optimized by incorporating incident radiation, temperature, and vegetation and topographic factors in mountainous areas.

In addition, the study found that five ET products exhibited varying biases across different climate zones. All ET products demonstrated a consistent tendency to underestimate in arid and semi-arid climate zones, whereas overestimation was prevalent in humid and semi-humid zones (see Figure 6). This deviation may be attributable to heterogeneity in vegetation types, precipitation, or soil moisture across different study regions. Therefore, climate factors should also be incorporated when refining the ET model.

### 5.3. Applications Recommendation of ET Products

The proliferation of ET products has enhanced our comprehension of the ET process, yet it also presents challenges in selecting the appropriate product for application. Based on the results of this study, we provide recommendations for applying these five products across different regions and ecosystems.

As shown in Table 4, on the TP, PML_V2 has the highest accuracy in grasslands and wetlands, ETMonitor achieves the highest accuracy in barren lands, and GLASS performs well in forests. Therefore, PML_V2 is recommended for vegetation phenology monitoring, ETMonitor for water resource management over areas with sparse vegetation, and GLASS for forest fire warning. In the YGP, PML_V2 achieves the highest accuracy in croplands, while GLASS performs best in forests. Consequently, PML_V2 is recommended for agricultural irrigation guidance, whereas GLASS is advised for forest drought monitoring applications in the YGP. Additionally, MOD16 is found to be more suitable for localized regional applications, such as forest fire monitoring on the TP and agricultural irrigation guidance in the YGP.

In Region I, PML_V2 performs exceptionally well in forests, grasslands, and croplands; ETMonitor achieves the highest accuracy in wetlands; GLASS performs best in forests; and SSEBop performs well in barren areas. Therefore, PML_V2 is suitable for vegetation phenological analysis; ETMonitor is recommended for water resource management applications; GLASS is recommended for forest fire monitoring. In Region II, PML_V2 is deemed suitable for forest drought monitoring, while GLASS is appropriate for a wide range of wetland water resource management. Although SSEBop offers moderate accuracy across various regions, its global coverage and long-term time series make it suitable for global, long-term studies of hydrological analysis.

### 5.4. Comparison with Existing Study and Limitations of This Study

#### 5.4.1. Comparison with Existing Study

Recent studies have extensively evaluated ET products globally [83,87,88], yet few have investigated their performance and driving factors across distinct geographical regions. Comparing our findings with recent literature reveals both consistencies and novel insights. Zhao et al. [89] identified GLASS as highly accurate in the Three-River-Source Region, with solar radiation and LAI as primary drivers—a result consistent with our Geodetector findings for the TP. However, unlike their study, which was limited to three products and five factors, we assessed five products and investigated 14 factors. Similarly, Zheng et al. [47] and Tang et al. [77] reported high accuracy for PML_V2 and ETMonitor over the TP, while noting the poor performance of MOD16. Tang et al. [77] further identified Prec and LAI as dominant factors, aligning with our results; however, our study uniquely emphasizes the comparison of ET accuracy across different ecoregions and explores spatial heterogeneity in driving factors based on model input parameters. Unlike Jin et al. [4], who relied solely on cross-validation among products, we validated findings against in situ data. By evaluating five products under diverse geographical conditions, this study provides a valuable reference for selecting appropriate ET products and refining models for water resource management in varying terrains.

#### 5.4.2. Limitations of This Study

This study is subject to several limitations. (1) The validation of the five ET products using EC sites revealed significant disparities in site density and spatial distribution across regions. Specifically, the number of sites in the YGP (5 EC) and Region II (3 EC) was notably insufficient. Sparse EC observations can compromise the accuracy of such assessments [80]. Despite the limited number of EC stations in the YGP and Region II—which may constrain the reliability of the validation results to some extent—the findings still effectively capture the accuracy differences among the five ET products between plateau and plain regions in humid climates. Furthermore, significant variations in observation periods across the selected EC sites limit the precision of temporal ET analysis. Moreover, discrepancies in temporal scales can influence the validation outcomes for various ET products. Consequently, future research should diversify data sources and employ a time-weighted validation approach to yield more robust results. (2) The accuracy evaluation relied exclusively on in situ EC data, lacking validation at a regional scale. (3) The lack of energy-balance closure corrections at the EC sites inevitably introduced uncertainty into the ET product assessment. (4) This study utilized mean values from time-series data for key driving-factor analysis, failing to account for interactions and correlations among factors, as well as the drivers influencing the spatial patterns of ET. Future research will integrate the watershed water balance method or the generalized triangle method to assess product accuracy while also examining the spatial distribution of ET products, focusing on factors such as topography.

## 6. Conclusions

This study quantitatively evaluated the performance and dominant driving factors of five ET products across the plateau and plain regions of China from 2003 to 2020. Based on in situ EC observations, we conducted subregional and ecosystem-specific validation, along with a mechanistic analysis of ET drivers across typical terrain types. The key conclusions are as follows:

(1) The performance of ET products varied across ecosystems and regions. PML_V2 excelled in grasslands and wetlands on the TP, ETMonitor in barren areas, and GLASS in forests. In Region I, PML_V2 performed well in forests, grasslands, and croplands, while SSEBop was suitable for barren areas. In the YGP, PML_V2 was recommended for agricultural water management, and GLASS for forest drought monitoring. These findings highlight the importance of selecting ET products based on regional characteristics.

(2) The spatial distribution of ET products showed good consistency in lowland areas but significant discrepancies in plateau regions, particularly the TP. A time-series analysis revealed substantial differences in mean annual ET and its trends across regions. These findings underscore the critical need to optimize ET models based on specific regional characteristics to improve estimation accuracy.

(3) Dominant driving factors vary regionally: on the TP, thermal radiation factors predominate; the YGP is characterized by a combination of thermal radiation and moisture factors, while plains show a synergistic interaction among thermal radiation, moisture, and vegetation factors.

(4) Several limitations were identified, including an uneven spatial distribution of EC sites, scale mismatch between EC footprint and ET products, and lack of energy-balance closure at the EC station. Future research should employ multi-source validation strategies, incorporate vegetation and soil moisture data into P-M type models, and couple topography and vegetation data within energy-balance models to optimize algorithms and improve ET estimation accuracy. Incorporating topographic, climatic, and vegetation information through data assimilation methods could yield new ET products, enhancing accuracy in mountainous areas and providing robust data support for water resource management under climate change scenarios.

In conclusion, this study analyzed and evaluated five ET products under different geographical conditions, providing a reference for selecting appropriate ET products and refining models for water resource utilization under climate change in various terrain conditions.

## Figures and Tables

**Table 1:** Essential details of the 34 EC systems used in this study.

Study Area	Site ID	Name	Lat (°N)	Lon (°E)	Ele (m)	LCT	Time	Number Months	Sources
TP	SDL	Sidalong	38.43	99.93	3146	F	January 2020–December 2021	24	ITP
	SJCSP	Sanjiangyuan	35.25	100.70	3960	G	January 2012–December 2016	60	CF
	REG	Ruoergai	32.80	102.55	3500	G	May 2015–December 2020	68	CF
	ARS	A’rou	38.05	100.46	3033	G	January 2008–December 2022	168	HiW
	JYL	Jingyangling	37.84	101.12	3750	G	January 2018–December 2022	60	HiW
	DDS	Dadongshu	38.01	100.24	4148	G	January 2015–December 2022	96	HiW
	DSL	Dashalong	38.84	98.94	3739	G	January 2013–December 2022	120	HiW
	MAW	MAWORS	38.41	75.05	3647	G	January 2012–December 2021	96	ITP
	NAM	NAMORS	30.77	90.98	4730	G	January 2007–December 2021	168	ITP
	SET	SETORS	29.77	94.73	3327	G	January 2007–December 2021	156	ITP
	Ngoring	Ngoring	34.91	97.55	4280	G	January 2012–December 2020	108	ITP
	Maqu	Maqu	33.92	102.15	3434	G	January 2014–December 2019	72	ITP
	TGL	Tanggula	33.03	92.01	5150	W	November 2019–April 2021	18	ITP
	SGH	Suganhu	38.99	94.12	2823	W	December 2019–December 2021	25	ITP
	NASED	NASED	33.39	79.70	4270	B	January 2010–December 2021	144	ITP
	QOMS	QOMS	28.36	86.95	4276	B	April 2007–December 2021	177	ITP
YGP	JFS	Jianfoshan	29.02	107.15	1543	F	January 2020–December 2021	24	CF
	ALS	Ailaoshan	24.54	101.03	2450	F	January 2009–December 2013	60	CF
	YJ	Yuanjiang	23.48	102.18	481	F	May 2013–December 2015	32	CF
	GZPD	Puding	26.60	106.32	1166	C	January 2015–December 2019	60	CF
	XSBN	Xishuangbannan	21.93	101.25	580	F	June 2010–December 2014	55	CF
Region I	NMDM	Neimengdamao	41.64	110.33	1409	D	January 2015–December 2018	48	CF
	NMDL	Neimengduolun	42.05	116.28	1324	G	January 2006–December 2015	120	CF
	HXLZ	Linze	39.33	100.14	1350	C	January 2012–December 2015	48	CF
	HB	Haibei	37.62	101.32	3216	G	January 2015–December 2020	72	CF
	PJSD	Panjin	40.93	121.96	2	W	January 2018–December 2020	36	CF
	QY	Qingyang	35.67	107.85	1297	C	January 2019–December 2021	36	CF
	HZZ	Huazhazi	38.77	100.32	1731	D	January 2012–December 2022	132	HiW
	BTM	Baotianman	33.49	111.94	1411	F	January 2017–December 2018	24	CF
	XLD	Xiaolangdi	35.03	112.47	410	F	January 2016–December 2017	24	CF
	YKe	Yingke	38.86	100.41	1519	C	January 2008–December 2011	48	CF
Region II	DTH	Dongtinghu	29.49	113.05	29	W	June 2014–December 2016	31	CF
	QYZ	Qianyanzhou	26.74	115.06	111	F	January 2003–December 2010	96	CF
	DHS	Dinghushan	23.17	112.53	14	F	January 2003–December 2010	96	CF

Due to the XSBN location in the tropical rainforest mountains of Xishuangbanna Dai Autonomous Prefecture, Yunnan Province, this study classifies this site as part of the Yungui Plateau. F is forest, B is barren, C is cropland, D is desert, G is grassland, and W is wetland. CF represents ChinaFLUX, HiW represents HiWATER, and ITP represents ITPCAS.

**Table 2:** Information on five ET products.

ET Products	Spatial Resolution	Temporal Resolution	Time Span
MOD16	500 m	8 days	2001–present
GLASS	1000 m	8 days	2000–2023
PML_V2	500 m	Daily	2000–2020
SSEBop	1000 m	Monthly, Yearly	2003–present
ETMonitor	1000 m	Daily, Monthly	2000–2021

**Table 3:** Accuracy statistics for five ET products under different LCTs on the YGP and Region II.

Region	LCT	Precision Index	MOD16	GLASS	PML_V2	SSEBop	ETMonitor
YGP	forest	R^2^	0.46	0.53	0.30	0.25	0.26
		RMSE	35.13	23.66	27.64	32.09	31.02
		Bias	25.16	9.38	5.47	2.62	4.03
	cropland	R^2^	0.75	0.91	0.78	0.84	0.94
		RMSE	34.29	17.48	11.19	27.95	23.16
		Bias	30.81	12.21	7.21	14.24	17.16
Region II	forest	R^2^	0.47	0.75	0.68	0.66	0.7
		RMSE	36.77	19.76	19.22	25.21	27.59
		Bias	23.95	11.27	8.37	4.17	18.86
	wetland	R^2^	0.58	0.63	0.52	0.43	0.58
		RMSE	26.63	18.73	24.24	55.56	33.44
		Bias	11.72	−8.65	1.26	20.38	16.94

The units of RMSE and bias are mm/month. LCT stands for land cover type. In the YGP, the EC in forested areas includes JFS, ALS, YJ, and XSBN, totaling 171 months of data; the station in the cropland area is GZPD, with 60 months of data. In Region II, the stations in the forest area include QYZ and DSH, spanning 192 months; the station in the wetland area is DTH, covering 31 months.

**Table 4:** Recommended applications for ET products across different regions and LCT.

LCT/Region	TP	YGP	Region I	Region II
Forest	GLASS	GLASS	PML_V2	PML_V2
Grassland	PML_V2	-	PML_V2	-
Wetland	PML_V2	-	ETMonitor	GLASS
Barren	ETMonitor	-	SSEBop	-
Cropland	-	PML_V2	PML_V2	-
Entire region	ETMonitor	GLASS	PML_V2	GLASS

The symbol “-“ denotes that, owing to the absence of validation data, the recommended ET product cannot be determined.

## Data Availability

The original data presented in the study are publicly available. The EC data of ChianFLUX are accessible at http://www.chinaflux.org, accessed on 30 April 2026. The EC data of HiWATER are accessible at http://www.chinaflux.org, accessed on 30 April 2026. The EC data of ITPCAS are accessible at https://doi.org/10.11888/Terre.tpdc.301321, accessed on 30 April 2026. The MOD16 data are accessible at https://www.ntsg.umt.edu, accessed on 30 April 2026. The GLASS data are accessible at https://glass.hku.hk, accessed 30 April 2026. The PML_V2 data are accessible at https://doi.org/10.11888/Terre.tpdc.272389, accessed on 30 April 2026. The SSEBop data are accessible at https://earlywarning.usgs.gov, accessed on 30 April 2026. The ETMonitor data are accessible at https://data.apps.fao.org/catalog/dataset/758af7e7-0d91-4021-9b66-4af7e80a0366 (accessed on 30 April 2026). and https://www.tpdc.ac.cn/zhhans/data/c284bd88-7694-4577-9cbb-02684bd940ff/, accessed on 30 April 2026. CFMD is accessible at https://dx.doi.org/10.11888/Atmos.tpdc.302088, accessed on 30 April 2026.

## Abbreviations

The following abbreviations are used in this manuscript:

BMA	Bayesian model averaging
BR	Bowen ratio
CMFD	China Meteorological Forcing Dataset
DLR	Downward longwave radiation
EC	Eddy covariance
ECM	Extended collocation method
ERA5	Fifth-generation ECMWF atmospheric reanalysis
ET	Evapotranspiration
GEE	Google Earth Engine
GLASS	Global Land Surface Satellite
GLEAM	Global Land Evaporation Amsterdam Model
HiWATER	Heihe Watershed Allied Telemetry Experimental Research
ITPCAS	Institute of Tibetan Plateau Research, Chinese Academy of Sciences
LASs	Large-aperture scintillometers
LAI	Leaf area index
LCT	Land cover type
LE	Latent heat
MOD16	MOD16A2GF Version 6.1
MMLST	Monthly Mean Land-Surface Temperature
NDVI	Normalized Difference Vegetation Index
Pa	Atmospheric pressure
PML_V2	Penman–Monteith–Leuning V2
Prec	Precipitation
R	Pearson correlation coefficient
R^2^	Coefficient of determination
RH	Relative humidity
RMSE	Root mean square error
SSD	Sunshine duration
SSEBop	Simplified Surface Energy Balance Operational ET
STD	Standard deviation
SWD	Downward shortwave radiation
Ta	Air temperature
TC	Triple collocation
TCH	Three-cornered hat
TP	Tibetan Plateau
VPD	Vapor pressure deficit
WS	Wind speed
YGP	Yunnan–Guizhou Plateau
